# Adverse reaction to metal debris with accompanying gout and amyloid deposits in hip arthroplasty

**DOI:** 10.1016/j.radcr.2022.11.079

**Published:** 2023-01-05

**Authors:** Love Shah, Joseph Zywiciel, Alexander Kui, Denton Connor, Cheryl Zhang, Maria M. Picken, Emad Allam

**Affiliations:** Loyola University Chicago and Loyola University Medical Center, 2160 S 1st Ave, Maywood, IL 60153, USA

**Keywords:** Adverse reaction to metal debris, Adverse local tissue reaction, Pseudotumor, Metal-on-metal hip arthroplasty, Gout, Amyloid, MoM, metal-on-metal, ARMD, adverse reaction to metal debris, MAVRIC, multi-acquisition variable resonance image combination, MPO, myeloperoxidase, p-ANCA, perinuclear-antineutrophil cytoplasmic antibodies, BMI, body mass index, CRP, C-reactive protein, ESR, erythrocyte sedimentation rate, RBC, red blood cell, WBC, white blood cell

## Abstract

Adverse reaction to metal debris (ARMD) is a known complication of metal-on-metal hip arthroplasty. There has been one previously reported case of ARMD with concomitant gout in the setting of a hip arthroplasty. We report a case of ARMD with accompanying monosodium urate crystals as well as amyloid deposition in the hip of a patient who had undergone a metal-on-metal hip arthroplasty. This is the only published case to date of these 3 conditions co-existing, although it is possible that the incidence is higher since these require special diagnostic tests that are not routinely performed. It is postulated that these entities are biochemically associated with each other rather than being purely coincidental.

## Background

Historically, metal-on-metal (MoM) implants were considered a biomechanically favorable option for patients requiring total hip arthroplasty implants: they have less volumetric wear and low failure rates due to wear-induced osteolysis, and have the ability to contain larger femoral heads that increase stability [1]. However, over time MoM implants started to show increasing revision rates attributed to the release of metal particles in the periprosthetic space creating a spectrum of findings that are termed adverse reaction to metal debris (ARMD) [2]. Diagnosis of ARMD related prosthesis failure is typically indicated by an abnormally elevated serum cobalt level, but concomitant diagnoses may also be identified and may complicate the interpretation of abnormal lab results. One previous case report identified 2 patients with concurrent ARMD and crystalline arthropathy (one patient with monosodium urate and the other with calcium pyrophosphate crystals aspirated), emphasizing the complexity of symptomatic ARMD while concluding that the crystalline arthropathy was not the primary cause of symptomatic hip inflammation [3]. Furthermore, we were unable to find a case of ARMD with concurrent amyloid deposition in the literature. We present a unique case of ARMD with accompanying monosodium urate crystals and amyloid deposition in the hip of a patient who had undergone a MoM hip arthroplasty. This is the only published case to date of a patient with these 3 co-existing conditions.

## Case presentation

A 71-year-old male presented with left hip/groin pain. He had a history of a MoM left hip arthroplasty performed 13 years prior to presentation. The components were a Zimmer CLS #8 cementless femoral stem, 135-degree femoral neck, 50 mm head, plus 4 mm neck, and 56 mm Duracon acetabulum. He also had a right total hip arthroplasty that was placed 21 years prior. The arthroplasties were performed for osteoarthritis. He denied any recent trauma. He reported no fevers or chills. No neurologic symptoms were described. He had a history of MPO/p-ANCA positive vasculitis for which he was on low dose rituximab. He had no other relevant medical or surgical history. In particular, there was no history of gout or amyloidosis. His BMI was 27 kg/m^2^. There was no erythema or drainage from the surgical scar. He was able to walk without any assistive devices. Blood tests revealed elevated CRP and normal ESR. Further blood tests revealed elevated cobalt and negative chromium.

**Fig. 1:**
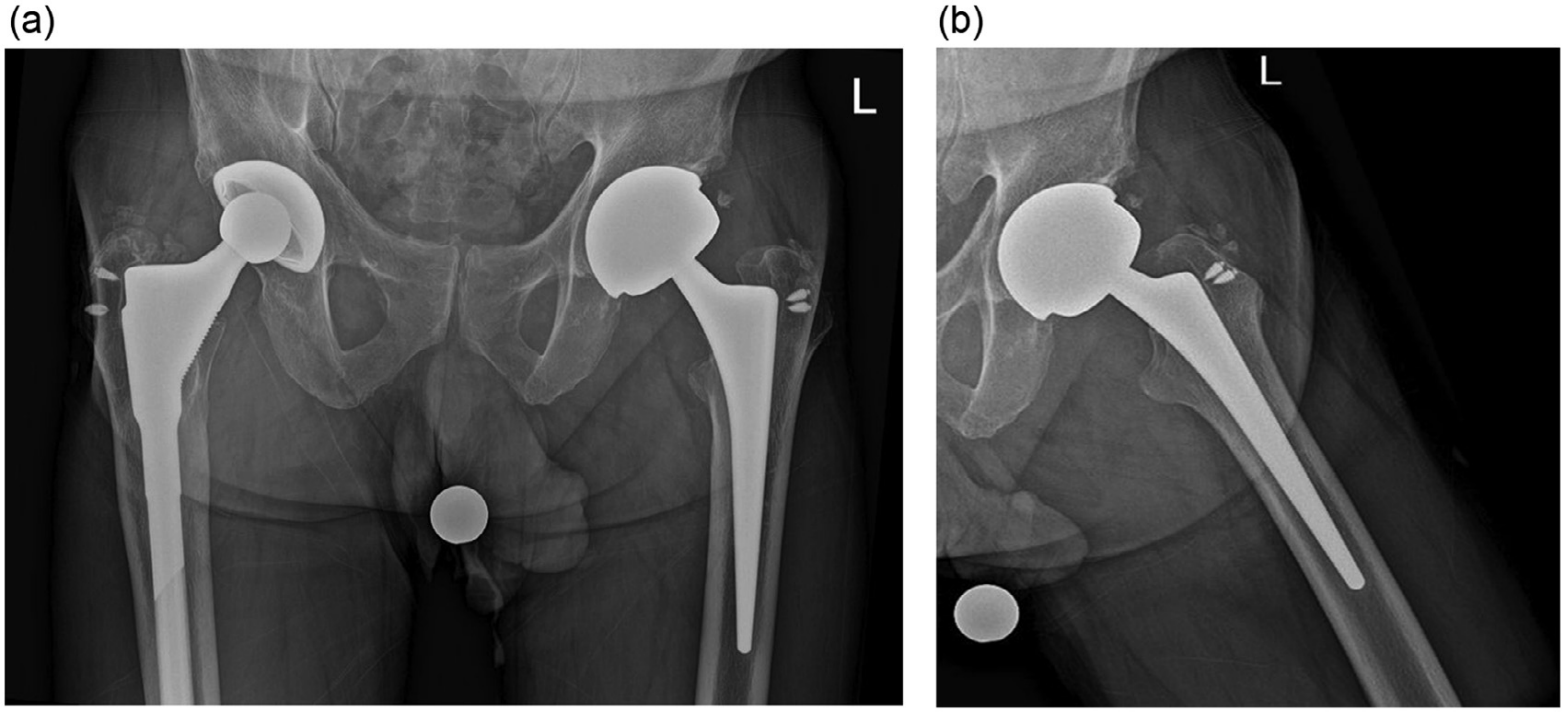
(a) Low-centered AP radiograph of the pelvis and (b) lateral radiograph of the left hip demonstrate a metal-on-metal left total hip arthroplasty. There is also a right total hip arthroplasty with a metal-on-polyethylene design. Suture anchors are present in the bilateral greater trochanters. No metallic debris is visible radiographically.

**Fig. 2:**
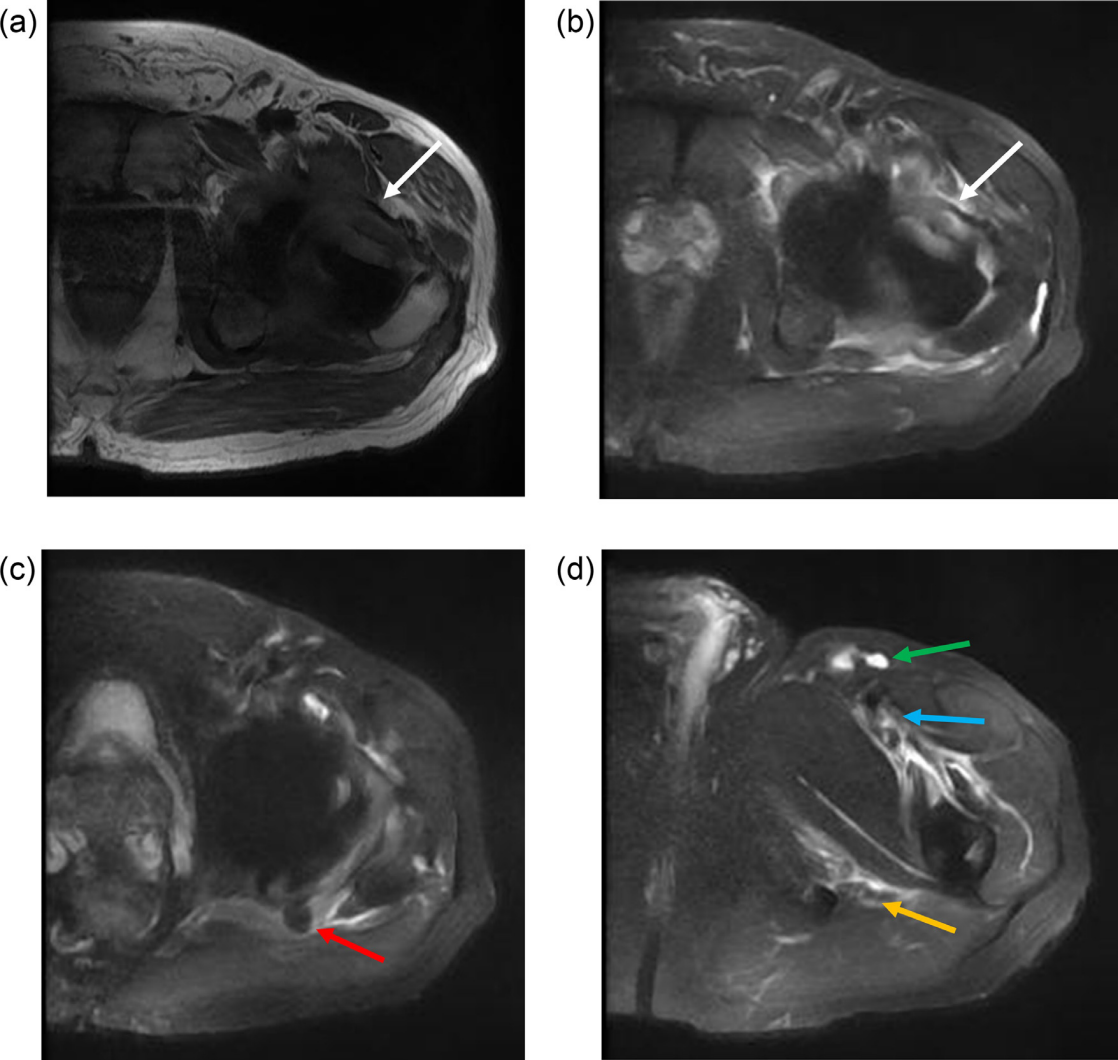
(a) Axial T1 and (b) axial STIR images from MRI of the left hip using a MAVRIC protocol for metal artifact reduction. These demonstrate distention of the hip joint capsule due to complex fluid/synovitis, best seen along the femoral neck anteriorly (white arrows). There is surrounding edema. (c) Axial STIR image shows a metallic particle posterior to the left hip (red arrow). (d) Axial STIR image shows edema-like signal around the left proximal femur extending to the femoral vessels (blue arrow) and sciatic nerve (orange arrow). Non-enlarged hyperintense left inguinal lymph nodes are noted (green arrow).

**Fig. 3:**
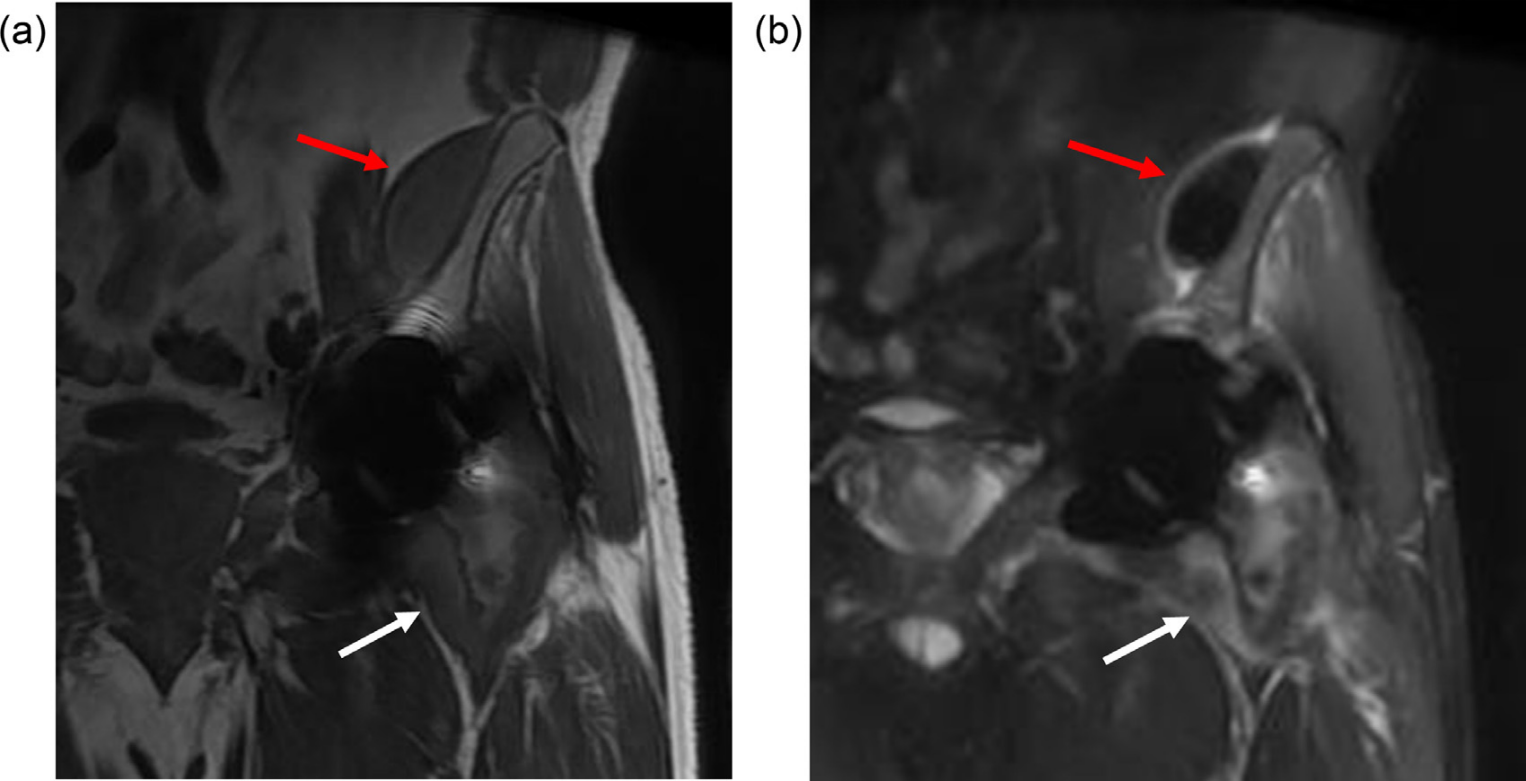
(a) Coronal T1 and (b) coronal STIR images from MRI of the left hip. These demonstrate a low signal intensity structure along the medial aspect of the left iliac bone, compatible with metallic debris (red arrows). The left hip arthroplasty is seen with distention of the joint capsule, particularly along the medial aspect of the femoral neck (white arrows).

**Fig. 4:**
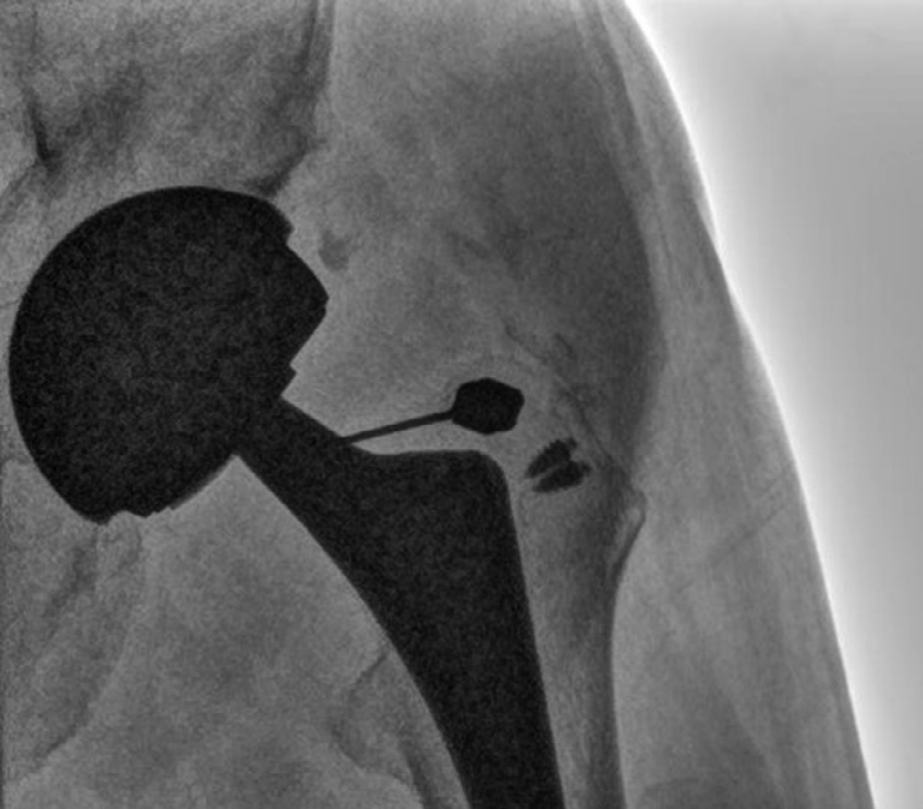
Intraprocedural image during fluoroscopically guided aspiration of the left hip showing the needle tip projecting at the neck of the femoral prosthesis.

**Fig. 5:**
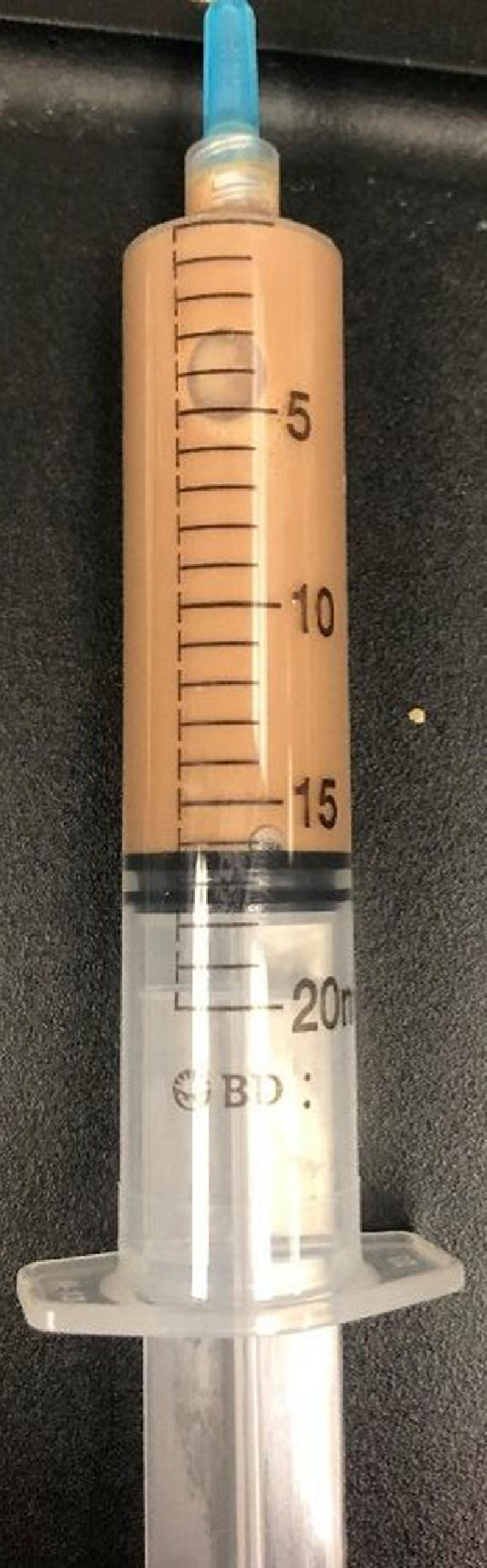
Aspiration of the left hip yielded 16 mL of cloudy brown fluid.

Radiographs demonstrated bilateral total hip arthroplasties with well-seated components (Fig. 1). No periprosthetic lucency or metallic debris was visible. Subsequent MRI with metal artifact reduction revealed distention of the left hip joint capsule with surrounding edema extending into the adjacent muscles and around the femoral vessels and sciatic nerve (Fig. 2). Prominent metallic debris was seen superiorly along the left iliac bone, presumed to have migrated there via the iliopsoas bursa (Fig. 3). Fluoroscopically-guided aspiration of the left hip with an 18-gauge needle yielded 16 mL of cloudy brown fluid (Figs. 4 and 5). The aspirated fluid was positive for cobalt and chromium, and was also positive for monosodium urate crystals. The synovial RBC count was 234,000 /μL and the WBC count was 11,650 /μL with differential unable to be performed due to degenerated cells. Aerobic and anaerobic cultures were negative for organisms. Findings were compatible with ARMD in the setting of a MoM hip arthroplasty.

**Fig. 6:**
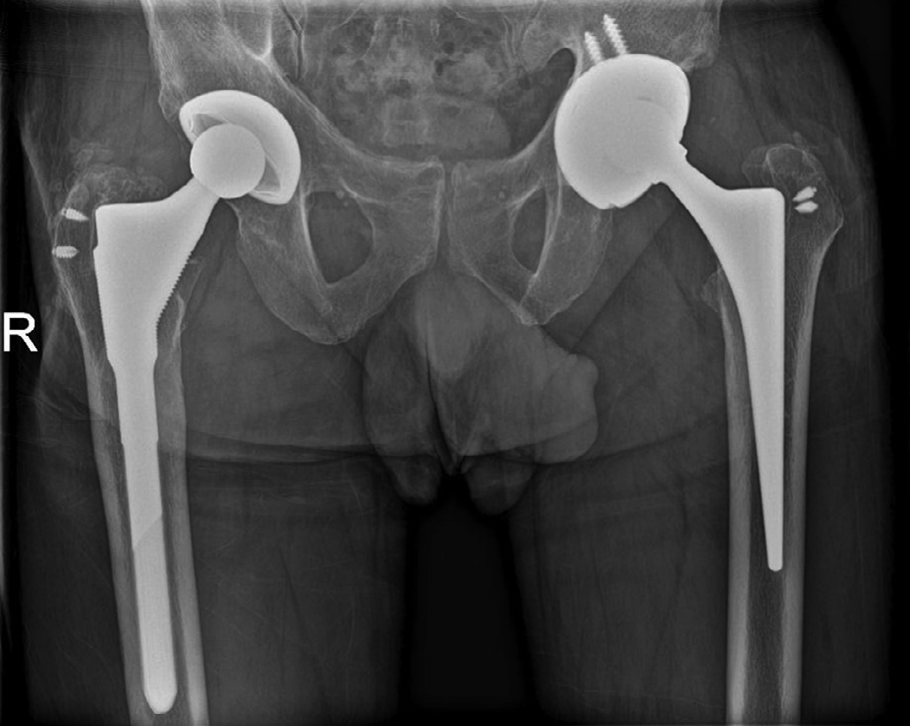
Post-operative low-centered AP radiograph of the pelvis demonstrates interval revision of the left total hip arthroplasty, now with a metal-on-polyethylene design. The right hip arthroplasty is unchanged.

**Fig. 7:**
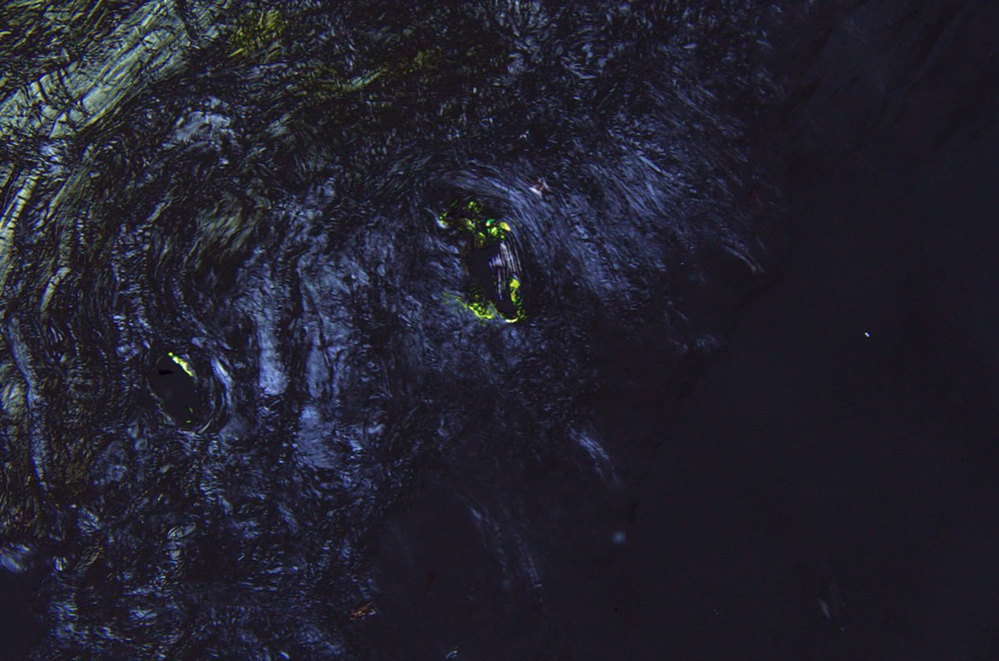
Congo red stained left hip joint capsule section examined under polarized light shows apple-green birefringence, indicating amyloid positivity. The amount of amyloid was small and not enough for sub-typing. Original magnification 200×.

The patient underwent revision of the left total hip arthroplasty, including the entire acetabular component and the femoral head with placement of a polyethylene liner (Fig. 6). Thick pseudocapsule formation, milky joint fluid, and sub-centimeter areas of osteolysis were noted at the time of surgery. Pathologic examination of the left hip joint capsule revealed focal deposits of amyloid by Congo red stain (Fig. 7). The post-operative course was complicated by capsular dehiscence and subcutaneous fluid collection.

## Discussion

ARMD is a known complication of MoM articulations, which generate metal ions and particulate debris from corrosive wear leading to the stimulation of immune responses. ARMD is also referred to as “adverse local tissue reaction” which is a term used to describe a plethora of histological findings associated with immune system activation [3], [4], [5]. The most common symptoms and physical exam findings in ARMD are hip/groin pain. Systemic symptoms such as vision loss, hearing loss, and cardiac problems are rare but can occur with long term metal toxicity. Blood tests commonly show elevated CRP and cobalt levels [6,7]. Synovial fluid analysis may serve as a useful screening tool for ARMD and other pathologies, and is important in ruling out infection [3]. Radiographs and MRI (optimized for metal artifact reduction) are the main imaging modalities used in the work-up of ARMD. Common MRI findings include increased joint fluid, synovial thickening, low signal intensity debris, soft tissue edema, and abductor disruption. Ultrasound and CT may also be used but have a relatively lower sensitivity/specificity when it comes to ARMD identification [4].

Crystal deposits are commonly seen in cases of crystalline arthropathy such as gout and pseudogout. These 2 conditions may present suddenly with accompanying swelling, pain, and stiffness around the affected joint [3]. The pathogenesis involves monosodium urate (gout) or calcium pyrophosphate (pseudogout) crystal deposits within a joint leading to the initiation of an acute inflammatory cascade [8]. Risk factors for crystalline arthropathy include age, diet, weight, family history, and recent surgical procedures. A common diagnostic procedure includes synovial fluid aspiration in which the presence of crystals can confirm crystalline arthropathy. Treatments are designed to focus on the reduction of inflammation while also working to lower the amount of uric acid in the blood [8].

There have been 2 documented cases where crystalline arthropathy has been seen in conjunction with ARMD, both of which were confirmed with synovial fluid aspiration. It is unclear if crystalline arthropathy may be a result or cause of ARMD. However, a possible etiology of the accompanying crystalline arthropathy includes the change in joint space pH (acidosis) due to ARMD, which can trigger the development/insoluble state of urate crystals leading to gout [3]. Crystalline arthropathy has been reported to resolve following MoM hip arthroplasty revision, further suggesting an association. While it is important to note that crystalline arthropathy is not reported as the cause of hip arthroplasty failures, it may play a role in the associated symptoms.

We report a case of ARMD with accompanying monosodium urate crystals as well as amyloid deposition in the hip of a patient who had undergone MoM hip arthroplasty. Amyloid arthropathy is a disease that is characterized by the deposition of insoluble protein fibrils in the tissue, most notably transthyretin and apolipoprotein A-I. However, some patients, similar to our patient, have unclassified amyloid owing to paucity of amyloid deposits [9]. Amyloid deposition around joint tissues and synovia can cause the affected joint to develop pain and edema which can be misdiagnosed as gout or rheumatoid arthritis [10]. Amyloid deposits are best identified on pathologic examination via Congo red stain examined under polarized light. Amyloid deposits consist of aggregates of certain proteins which are susceptible to misfolding with changes in pH. We hypothesize that the accompanying urate crystal deposits as well as the amyloid deposits occur due to a change in joint space pH. The acidic environment that develops from ARMD may predispose to urate crystal deposits; if the pH drops low enough it may also cause protein misfolding and amyloid formation within the joint space. In other words, both crystalline arthropathy and amyloid arthropathy may be downstream effects of ARMD in patients with MoM hip arthroplasties and should not be treated in isolation. It is advised to screen for both urate crystals and amyloid plaques when creating a treatment plan for ARMD.

## Conclusion

We presented a patient with a history of MoM hip arthroplasty who complained of acute hip pain. This patient was diagnosed with ARMD along with co-existing urate crystal formation found prior to revision surgery and amyloid deposits found post-operatively, a combination which has not been previously described. We report this case to alert the orthopedist that crystalline arthropathy and amyloid deposits may present at the same time as ARMD, potentially a result of alterations in pH due to ARMD. Serum and synovial laboratory tests along with pathological examination may be abnormal because of crystal or amyloid development. The orthopedist should be aware of these possible co-existing conditions; ARMD often predominates the clinical picture but these other processes could accentuate symptomatology and potentially complicate the post-operative course.

## Patient consent

Written informed consent for the publication of this case report was obtained from the patient.
